# Exosome-Mediated Transfer of ACE2 (Angiotensin-Converting Enzyme 2) from Endothelial Progenitor Cells Promotes Survival and Function of Endothelial Cell

**DOI:** 10.1155/2020/4213541

**Published:** 2020-01-18

**Authors:** Jinju Wang, Shuzhen Chen, Ji Bihl

**Affiliations:** Department of Pharmacology and Toxicology, Boonshoft School of Medicine, Wright State University, Dayton, OH 45435, USA

## Abstract

Angiotensin-converting enzyme 2 (ACE2) is an emerging cardiovascular protective target that mediates the metabolism of angiotensin (Ang) II into Ang (1–7). Our group has demonstrated that ACE2 overexpression enhances the function of endothelial progenitor cells (EPCs). Here, we investigated whether ACE2-primed EPCs (ACE2-EPCs) can protect cerebral microvascular endothelial cells (ECs) against injury and dysfunction in an *in vitro* model, with focusing on their exosomal and cytokine paracrine effects on endothelial mitochondria. Human EPCs were transfected with lentivirus containing null or human ACE2 cDNA (denoted as Null-EPCs and ACE2-EPCs, respectively). Their conditioned culture media, w/wo depletion of exosomes (ACE2-EPC-CM^EX-^, Null-EPC-CM^EX-^, ACE2-EPC-CM, and Null-EPC-CM), were used for coculture experiments. EC injury and dysfunction model was induced by Ang II before coculture. Apoptosis, angiogenic ability, mitochondrion functions (ROS production, membrane potential, fragmentation), and gene expressions (ACE2, Nox2, and Nox4) of ECs were analyzed. The supernatant was collected for measuring the levels of ACE2, Ang II/Ang-(1–7), and growth factors (VEGF and IGF). Our results showed that (1) ACE2-EPC-CM had higher levels of ACE2, Ang (1–7), VEGF, and IGF than that of Null-EPC-CM. (2) Ang II-injured ECs displayed an increase of apoptotic rate and reduction in tube formation and migration abilities, which were associated with ACE2 downregulation, Ang II/Ang (1–7) imbalance, Nox2/Nox4 upregulation, ROS overproduction, an increase of mitochondrion fragmentation, and a decrease of membrane potential. (3) ACE2-EPC-CM had better protective effects than Null-EPC-CM on Ang II-injured ECs, which were associated with the improvements on ACE2 expression, Ang II/Ang (1–7) balance, and mitochondrial functions. (4) ACE2-EPC-CM^EX-^ and Null-EPC-CM^EX-^ showed reduced effects as compared to ACE2-EPCs-CM and Null-EPCs-CM. In conclusion, our data demonstrate that ACE2 overexpression can enhance the protective effects of EPCs on ECs injury, majorly through the exosomal effects on mitochondrial function.

## 1. Introduction

It is well accepted that the loss of endothelium integrity leads to endothelial dysfunction [1]. Accumulating evidence has indicated that endothelial progenitor cells (EPCs) could be recruited from the bone marrow to modulate endothelial function [2] and reestablish endothelium integrity [3, 4]. Several mechanisms might contribute to the actions of EPCs on promoting endothelium repair, including the ability of EPC differentiation into endothelial cells (ECs) [5, 6] and their paracrine effects such as secretion of growth factors to promote angiogenesis [7].

The renin-angiotensin system (RAS) is an important regulator of cardiovascular homeostasis. Angiotensin II (Ang II), a major peptide of RAS, is implicated in vascular dysfunction, which is majorly related to Ang II-induced induction of reactive oxygen species overproduction and activation of the redox-dependent signaling cascades [8, 9]. Ang II also can promote osteoblast cell apoptosis via impairing mitochondrial function [10]. Angiotensin-converting enzyme 2 (ACE2), which cleaves Ang II into angiotensin 1-7 (Ang1-7), has been suggested to protect against vascular injury in the development of diabetes [11–13]. Our group has demonstrated that ACE2 overexpression enhances the function of EPCs from renin and angiotensinogen transgenic mice [14]. However, whether ACE2-primed EPCs (ACE2-EPCs) have protective effects on Ang II-injured ECs has not been discovered.

Extracellular microvesicles including EXs have been recognized as a novel way of intercellular communication. Moreover, increasing evidence suggests that stem cell-derived EXs could execute the beneficial effects of their parent cells in regenerative medicine [15]. Mitochondria are the bioenergetic and metabolic centers of eukaryotic cells. Damages to the mitochondria could result in cell apoptosis [16]. Previous evidence has shown that Ang II can reduce mitochondrial content and induce loss of mitochondrial membrane potential in ECs and skeletal muscle [17, 18]. Interestingly, several groups found that stem cell-derived-EXs could protect cells such as neurons and cardiomyocytes against ischemia-induced apoptosis [19, 20], although it was unclear whether such beneficial effects were related to mitochondria protection. Most recently, we found that EPC-derived exosomes (EXs) exhibited favorable effects on decreasing mitochondrial fragmentation of ECs suffered to hypoxia/reoxygenation injury, associating with the regulation of fission and fusion protein expressions [21]. Nevertheless, there is limited study investigating whether ACE2-EPCs could protect Ang II-injured ECs through their released EXs on mitochondrion.

In this study, we determined whether ACE2-EPCs can protect ECs against Ang II injury through exosomal effects on mitochondrion.

## 2. Materials and Methods

### 2.1. Cell Culture

Human EPCs were purchased from Celprogen (Torrance, CA) and cultured in complete growth medium (Celprogen; Torrance, CA) according to the manufacturer's protocol. The medium was changed every other day. Human brain ECs were purchased from Cell Systems (Kirkland, WA) and cultured with CSC complete medium (Cell Systems; Kirkland, WA) containing 10% serum, 2% human recombinant growth factors, and 0.2% antibiotic solution under standard cell culture conditions (5% CO2, 37°C). The medium was changed twice a week.

### 2.2. Cell Transfection

The ACE2 transfection was performed by following the company protocol. Human EPCs were cultured to 60-70% confluence and cultured with 0.5 ml of complete optimal medium (with serum and antibiotics if required) at 37°C with 5% CO2 overnight before transfection. On the second day, the growth media were removed and replaced with 0.5 ml of the polybrene-media-mix (8 *μ*g/ml, Applied Biological Materials Inc., Richmond, BC, Canada). ViralPlus transduction enhancer G698 was used at 1 : 100. Then, the lentivirus with green fluorescent protein (GFP) reporter containing null or human ACE2 cDNA (denoted as Null-EPCs and ACE2-EPCs) (10 nM, Applied Biological Materials Inc.) was added into the cells overnight. The following day, cells were split at 1 : 3 or 1 : 5 (depending on the growth rate of your target cells) and continue incubating for 48 hours in complete media. EPCs cultured in complete culture medium served as the control. The efficiency of ACE2 transfection into EPCs was observed under a fluorescence microscope (EVOS; Thermo Fisher Scientific) and determined by quantitative RT-PCR and western blot analyses. Transduction efficiency (the percentage of GPF-expressing cells) was quantified by direct counting using an optical grid.

### 2.3. Animals

Adult (8–10 weeks of age; weight ranges from 25 to 32 g, male/female: half/half) renin transgenic (R+) mice and their age-matched controls (WT) with C57BL/6J genetic background were used for EC isolation. Totally, 10 of WT and 20 of R+ mice were used. The ECs isolated from each mouse could be used for all the assays. The strains were maintained in our laboratory. Mice were maintained in a 22°C room with a 12-hour light/dark cycle and fed with standard chow and drinking water ad libitum. All experimental procedures were approved by the Wright State University Laboratory Animal Care and Use Committee and were in accordance with the Guide for the Care and Use of Laboratory Animals issued by the National Institutes of Health.

### 2.4. Isolation and Depletion of EXs from EPC Culture Medium (EPC-CM)

EPC-CM were collected by centrifuging at 300 g and 15 mins to remove cells and cell debris. The supernatants were then centrifuged at 2000 g and 30 mins to remove cell debris. To collect/deplete EXs, the supernatant was ultracentrifuged at 170,000 g to collect/remove EXs. The speed we used here for depleting EXs is based on our previous publication of isolating EXs [22]. The media from ACE2-EPCs and Null-EPCs with/without EX depletion, named as ACE2-EPC-CM^EX-^, Null-EPC-CM^EX-^, ACE2-EPC-CM, and Null-EPC-CM, respectively, were used for coculture with Ang II-injured ECs. The EXs collected from the EPCs and ACE2-EPCs were identified as EPC-EXs and ACE2-EPC-EXs and used for coculture with ECs from R+ mice.

### 2.5. Isolation of ECs from the Aorta of WT and R+ Mice

The initial harvesting of the ECs was as previously described [23]. The aorta collected from the mice was cut into small pieces, coronally, and then placed lumen-side-down onto the matrix and placed in a 37°C, 5% CO2 humidified incubator for 36 hr. One ml of medium was then applied/well. After 7-10 days, once visible outgrowth from the tissue was observed, the aortic segment and medium were removed. When cells were confluent, the media was removed, and the cells washed with sterile PBS. Four ml of 0.3% collagenase I was added to the cells and allowed to incubate at 37°C for 30-40 min. The cells were then diluted with media, centrifuged, and washed with PBS containing 1% FBS. A 30 *μ*m preseparation filter (MACS, Miltenyi Biotec GmbH) was washed with the same buffer 3x. The cells were resuspended in 0.5 ml of PBS containing 1% FBS and passed through the preseparation filter to remove clumps. The cells were then washed 2x with PBS containing 1% FBS. Rat anti-mouse CD105 (10 *μ*g/ml, PharMingen, San Diego, CA) and rat anti-mouse CD106 (10 *μ*g/ml, PharMingen) were added to the cells. The cells were incubated on ice for 1 hr and then washed with RPMI medium, and resuspended in an equal volume of PBS/FBS and MACS goat anti-rat IgG microbeads (100 *μ*l of the bead suspension/10^7^ cells). The cells and beads were incubated at 4°C for 30 min. During the incubation, the MiniMACS Separation Unit was attached to the MACS multistand and the MS Separation column placed in the MiniMACS separation unit. The column was washed with 0.5 ml of PBS/1% FBS twice. The cells were then washed once with PBS/1% FBS, resuspended in degassed PBS/FBS, and then loaded onto the column. Cells that were positive for CD105 and CD106 were retained on the column. The column was then removed from the separator and the cells were collected by centrifugation, and resuspended in complete media, and plated onto collagen-coated flasks.

### 2.6. Coincubation Experiments

The EC injury model was induced by Ang II as previously described [24]. In brief, ECs were cultured in normal culture medium with Ang II (10^−6^ M, Sigma-Aldrich, St. Louis, MO).

Ang II-injured ECs were divided into five coculture groups: vehicle (EC culture medium only), ACE2-EPC-CM^EX-^, Null-EPC-CM^EX-^, ACE2-EPC-CM, and Null-EPC-CM.

The ECs isolated from the R+ mice were divided into six coculture groups: vehicle, EPCs, ACE2-EPCs, EPC-EXs, ACE2-EPC-EXs, and ACE2-EPC-EXS+DX600. The concentration of EPC-EXs (50 *μ*g/ml) was determined based on our previous study [21]. The concentration for ACE2 inhibitor (DX600) was 1 *μ*M [21]. After 24 hr of coculture, ECs were then used for various tests as described below.

### 2.7. Quantitative RT-PCR Analysis

The levels of ACE2 in EPCs transduced with Lenti-ACE2 were determined using a real-time RT-PCR method. EPC total mRNAs were isolated using the RNeasy Mini kit and reverse-transcripted with the high-capacity cDNA archive kit (Qiagen). Real-time PCR was run using SYBR Green reagents (Qiagen). The primer sequences for ACE2 were 5′- AAGCTAGCATAGCCAGGTCCTCCTGGCTCCTTC-3′ and 5′- AAGTCGACCTAAAAGGAAGTCTGAGCATCATCACTG-3′. *β*-Actin was chosen as the housekeeping gene for normalizing the data of gene expression. The mRNA level of ACE2 in EPCs from the control group was defined as 100%.

### 2.8. ACE2 Activity Assay

ACE2 activity was measured in cell lysates by using the ACE2 Activity Assay Kit (Fluorometric) (BioVision Inc., Milpitas, CA). One unit of ACE2 activity was the amount of enzyme that catalyzes the release of 1 nmol of MCA-Standard Curve per min from the substrate and were normalized for total protein or volume of the medium. The data are presented as the folds of the arbitrary fluorescence units (AFU) of WT.

### 2.9. Apoptosis Assay

After 24 hr of coculture, ECs were detached for apoptosis assay by using the FITC Annexin V apoptosis detection kit (BD Biosciences, CA) and analyzed by flow cytometry. The percentage of apoptotic cells was calculated as annexin V+/PI − cells/total cells × 100%.

### 2.10. The Angiogenic Functional Assays of ECs

The tube formation assay was conducted using the *in vitro* angiogenesis assay kit (Chemicon, Rosemont, IL) and the migration ability of ECs was evaluated using the Boyden chamber method as we previously reported [14].

### 2.11. ELISA Assays

The levels of Ang II and Ang (1–7) were determined by commercial kits (Enzo Life Sciences, Farmingdale, NY) per our previous publication [25]. The levels of vascular endothelial growth factor (VEGF) and insulin growth factor (IGF) in the cell culture condition medium were determined by commercial ELISA kits (R&D System, Minneapolis, MN).

### 2.12. Analysis of Mitochondrial Membrane Potential

The mitochondrial membrane potential (MMP) of ECs treated with various types of EPC-EXs was measured using the lipophilic cationic dye JC-1 (1 : 1000, Invitrogen) per our publication [21]. In brief, after 24 hr of treatment, ECs were washed with PBS and incubated with 1 ml of medium containing JC-1 staining probe (2 *μ*M final concentration) for 20 mins at 37°C. The cells were then washed with PBS and observed under a fluorescence microscope (EVOS; Thermo Fisher Scientific). The level of cellular fluorescence intensity was analyzed by Image J (NIH, Bethesda, MD). The relative MMP was calculated as the ratio of J-aggregate to monomer (590/520 nm). Values are expressed as fold increases in the ratio of J-aggregate to monomer fluorescence relative to control cells. ECs cultured with CSC medium were used as controls. The original images were all taken with the same gain/intensity with the same threshold. The people who did the analysis was blinded to the grouping information.

### 2.13. Measurement of ATP Level

Cellular ATP levels were measured using an ATP Assay Kit (Promega, WI, USA) according to the manufacturer's instruction as previously reported [21]. Briefly, the proteins of ECs treated with various types of EPC-EXs were harvested and determined by the BCA assay (Thermo Scientific). In 6-well plates, 200 *μ*l protein supernatant was mixed with 100 *μ*l ATP detection working solution. The luminance (RLU) was measured by a fluorescence microplate reader (Thermo Fisher Scientific, Varioskan Flash, USA). The total ATP levels were expressed as nmol/mg protein. The relative ATP level was calculated according to the following formula: relative ATP level = ATP value/protein concentration.

### 2.14. Assessment of Mitochondrial Morphology

To assess mitochondrial morphology, ECs growing on coverslips were stained with MitoTracker as our previous report [21]. In brief, cells were preincubated with MitoTracker Green FM (100 nM; Invitrogen) for 15 mins and then fixed in 4% paraformaldehyde (PFA) for 10 mins at RT. The nucleus was then counterstained with 4′,6-diamidino-2-phenylindole (DAPI). Mitochondrial morphology was observed under 60x oil immersion objective of a confocal microscope (Olympus FV-1000, Japan). Digital images were subjected to a convolve filter through the ImageJ software (NIH) to isolate and equalize fluorescent pixels in image [26]. Briefly, after thresholding, individual particles (mitochondria) were analyzed for circularity (4p′area/(perimeter^2^)) and lengths of major and minor axes. From these values, we calculated the form factor (FF; the reciprocal of circularity value) and aspect ratio (AR; major/minor). Both FF and AR have a minimum value of 1 when a particle is a small perfect circle and the values increase as the shape becomes elongated. A minimum of 100 cells from 10 random microscopic fields (60x) was assessed for mitochondrial fragmentation per coculture group in each independent experiment. Four independent experiments were conducted in each group.

### 2.15. Western Blot Analysis

Proteins of EPCs after transfection and ECs after different treatments were extracted with cell lysis buffer (Thermo Fisher Scientific, Waltham, MA) supplemented with a complete mini protease inhibitor tablet (Roche, Basel, Switzerland). Then, the protein lysates were electrophoresed through SDS-PAGE gel and transferred onto PVDF membranes. The membranes were blocked with 5% nonfat milk for 1 hr at room temperature and incubated with primary antibody against ACE2 (1:1000, Cell Signaling Technology), Nox2 and Nox4 (1 : 1000, Abcam) or *β*-actin (1 : 4000; Sigma, St. Louis, MO) at 4°C overnight. On the next day, membranes were washed and incubated with horseradish-peroxidase-conjugated anti-rabbit or anti-mouse IgG (1 : 40000; Jackson Immuno Research Lab, West Grove, PA) for 1 hr at room temperature. Blots were developed with enhanced chemiluminescence developing solutions and images were quantified using ImageJ software.

### 2.16. Statistical Analysis

Data are expressed as the mean ± SEM. Multiple comparisons were analyzed by one- or two-way ANOVA followed by Tukey post hoc test. SPSS 23.0 statistical software was used for analyzing the data. For all measurements, *P* < 0.05 was considered statistically significant.

## 3. Results

### 3.1. ACE2-EPC-CM Had Higher Levels of ACE2, VEGF, and IGF than That of Null-EPC-CM

**Figure 1:**
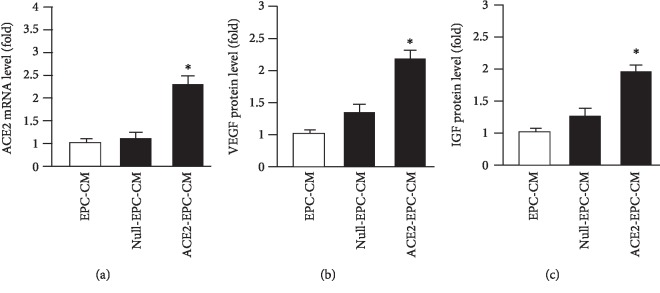
ACE2 transfection increased the mRNA level of ACE2 and the protein levels of VEGF and IGF. (a) qRT-PCR analysis of the ACE2 mRNA level in the three types of CM. (b, c) ELISA analysis of VEGF and IGF protein levels in the three types of CM. ^∗^*P* < 0.05 vs. EPC-CM; ^#^*P* < 0.05 vs. Null-EPC-CM. *N* = 6/group.

In order to determine whether ACE2 transfection could affect the secretion of ACE2 and growth factors of EPCs, we collected the CM of ACE2-EPCs and measured the levels of ACE2 mRNA and VEGF as well as IGF by qRT-PCR and ELISA. As revealed by the qRT-PCR data (Figure 1(a)), there was no significant difference in the mRNA level of ACE2 between EPC-CM and Null-EPC-CM, while it was significantly increased in ACE2-EPC-CM. Similarly, the protein levels of both VEGF and IGF were substantially increased in ACE2-EPC-CM (Figure 1(b)).

### 3.2. Ang II-Induced EC Apoptosis and Dysfunction, Accompanied by ACE2 Downregulation, a Decrease of MMP, and Increase of Mitochondrion Fragmentation

**Figure 2:**
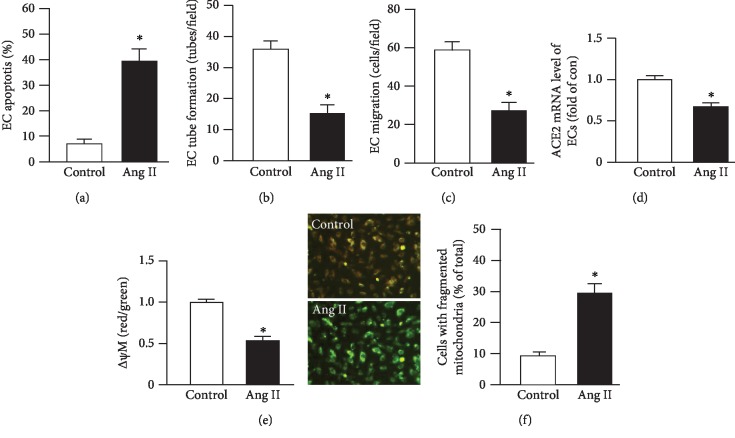
Characterization of Ang II-induced EC injury model. (a) EC apoptosis. (b, c) Tube formation and migration abilities. (d) ACE2 mRNA level in ECs. (e) Representative JC-1 staining images and summary data of *Δψ*M in ECs. *Δψ*M: mitochondrion membrane potential (MMP, red/green). Bar: 50 *μ*m. (f) Summary data showing the percentage of cells with fragmented mitochondria. ^∗^*P* < 0.05 vs. control. *N* = 6/group.

As shown in Figure 2(a), Ang II induced a significant increase of EC apoptosis as compared to that of control cells. Meanwhile, we found that the tube formation and migration abilities of ECs were compromised by Ang II as evidenced by fewer tubes formed in each field and fewer cells migrate through the Boyden chamber (Figures 2(b) and 2(c)).

Besides, our data (Figure 2(d)) showed that the ACE2 mRNA level was significantly decreased (~30% of the control) in Ang II-injured ECs. As revealed by Jc-1 staining, the MMP was decreased by about 50% by Ang II as compared to that of the control cells (Figure 2(e)). According to the data of MitoTracker Green FM staining, a larger percentage of ECs (~30% of cells) have fragmented mitochondria than that of control cells (Figure 2(f)).

These data suggest that Ang II-induced apoptotic cell death and dysfunction were associated with the compromised mitochondrion function.

### 3.3. ACE2-EPC-CM Was More Effective than Null-EPC-CM on Decreasing Ang II-Induced EC Apoptosis and Dysfunction

**Figure 3:**
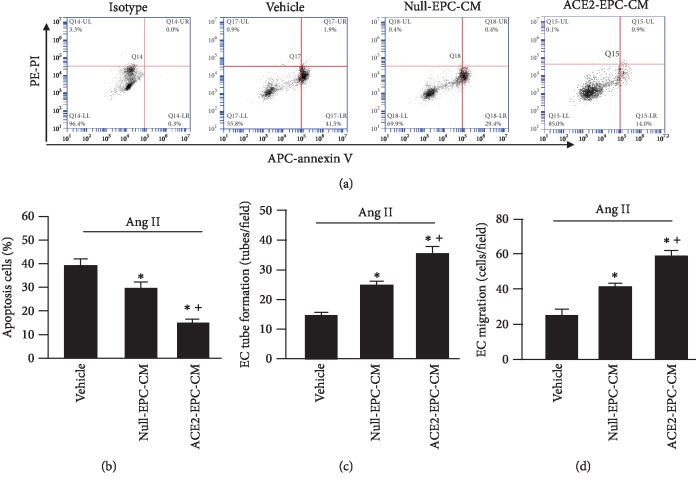
ACE2-EPC-CM decreased Ang II-induced EC apoptosis and dysfunction. (a) Representative flow plots of EC apoptosis. (b) Summarized data of EC apoptosis. (c) Tube formation. (d) Migration. ^∗^*P* < 0.05 vs. vehicle; ^+^*P* < 0.05 vs. Null-EPC-CM. *N* = 6/group.

After 24 hr coculture, we collected ECs and conducted an apoptotic assay. Our data (Figures 3(a) and 3(b)) showed that the percentage of early apoptotic ECs was lower after Null-EPC-CM treatment than that of the vehicle group (EC culture medium only). ACE2-EPC-CM coculture further decreased Ang II-induced apoptotic cell death.

To assess the angiogenic ability of ECs, we measured their tube formation and migration abilities. As expected, Null-EPC-CM could improve the angiogenic functions compromised by Ang II, which was prominently improved by ACE2-EPC-CM (Figures 3(c) and 3(d)).

### 3.4. ACE2-EPC-CM Upregulated ACE2 Level and Improved Ang II/Ang (1–7) Balance, Whereas ACE2-EPC-CM^EX-^ Reduced These Effects

**Figure 4:**
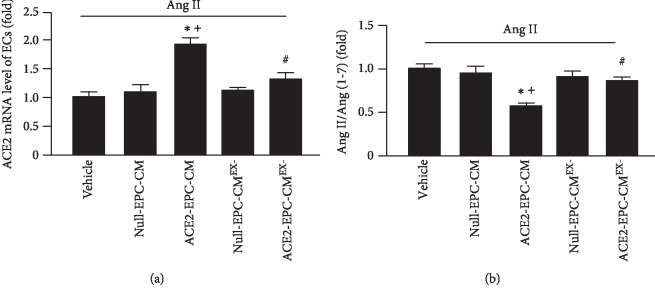
ACE2-EPC-CM upregulated ACE2 level and improved Ang II/Ang (1–7) balance on Ang II-induced ECs. (a) ACE2 mRNA level of ECs. (b) The ratio of Ang II/Ang (1–7). ^∗^*P* < 0.05 vs. vehicle; ^+^*P* < 0.05 vs. Null-EPC-CM; ^#^*P* < 0.05 vs. ACE2-EPC-CM. *N* = 6/group.

We performed qRT-PCR and ELISA to assess the levels of ACE2, Ang II, and Ang (1–7), respectively. Our data (Figure 4(a)) revealed that the mRNA level of ACE2 was not significantly changed by Null-EPC-CM treatment, but it was remarkably raised by ACE2-EPC-CM treatment. In order to investigate whether exosomes are responsible for such an effect, we centrifuged the CM to remove exosomes and then performed the coculture experiment. We found that there was no significant difference in the ACE2 mRNA level in Ang II-injured ECs treated by Null-EPC-CM and Null-EPC-CM^EX-^, whereas there was a significant decrease of ACE2 mRNA level in Ang II-injured ECs treated by ACE2-EPC-CM^EX-^ as compared to that of cells treated by ACE2-EPC-CM.

According to ELISA analysis (Figure 4(b)), the ratio of Ang II over Ang (1–7) in Ang II-injured ECs was remarkably decreased by ACE2-EPC-CM, whereas such effect was reduced by ACE2-EPC-CM^EX-^.

All of these data suggest that exosomes are responsible for ACE2-EPC-CM-induced ACE2 upregulation and improvement of Ang II/Ang (1–7) balance in Ang II-injured ECs.

### 3.5. ACE2-EPC-CM Was More Effective than Null-EPC-CM on Increasing MMP and ATP Level and Decreasing Mitochondrion Fragmentation, Whereas ACE2-EPC-CM^EX-^ and Null-EPC-CM^EX-^ Reduced These Effects

**Figure 5:**
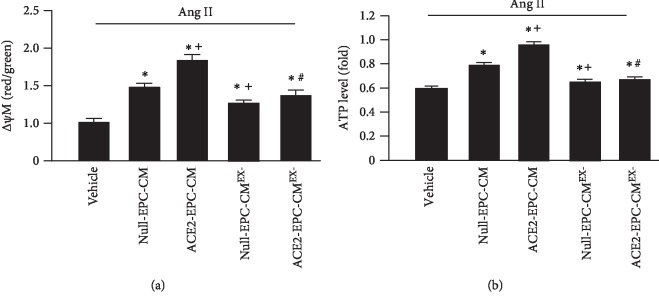
ACE2-EPC-CM increased MMP and ATP production. (a) The change of MMP in ECs after coincubation with different CM. (b) The ATP levels in ECs after different treatments. ^∗^*P* < 0.05 vs. vehicle; ^+^*P* < 0.05 vs. Null-EPC-CM; ^#^*P* < 0.05 vs. ACE2-EPC-CM. *N* = 6/group.

To assess the MMP of Ang II-injured ECs, we used JC-1 staining. Summarized data in Figure 5 showed that 24 hr treatment with Null-EPC-CM increased MMP (*Δψ*M, red/green) and the APT level of ECs, which was further increased by ACE2-EPC-CM treatment. There was no significant difference in MMP between Null-EPC-CM and Null-EPC-CM^EX-^. However, ACE2-EPC-CM^EX-^ reduced ACE-EPC-CM-induced increase of MMP.

**Figure 6:**
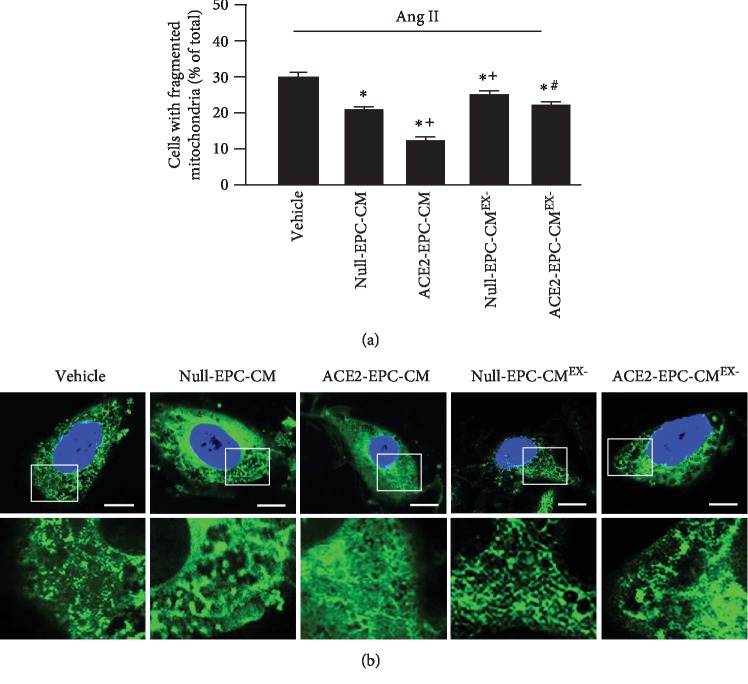
ACE2-EPC-CM decreased mitochondrion fragmentation. (a) Summary data showing the percentage of cells with fragmented mitochondria. (b) Representative confocal images showing the morphology of the mitochondrion. Enlarged images are magnifications of the mitochondria at the indicated area. Bar: 10 *μ*m. ^∗^*P* < 0.05 vs. vehicle; ^+^*P* < 0.05 vs. Null-EPC-CM; ^#^*P* < 0.05 vs. ACE2-EPC-CM. *N* = 6/group.

As shown in Figure 6, the representative pictures show the mitochondrial fragmentation (green) after different treatments. The summarized results showed that ECs exposed to Ang II (vehicle) exhibited a higher percentage of fragmented mitochondria in comparison to cells treated by Null-EPC-CM. What's more, ACE2-EPC-CM further decreased Ang II-induced mitochondrial fragmentation. Similarly, Null-EPC-CM^EX-^ did not alter the effect of Null-EPC-CM, but ACE2-EPC-CM^EX-^ significantly reduced ACE-EPC-CM-induced improvement of mitochondrial morphology.

Taken together, ACE2-EPC-CM had better efficient in improving Ang II-induced mitochondrial dysfunction and abnormality of mitochondrial morphology, which might be modulated by exosomes.

### 3.6. ACE2-EPC-CM Was More Effective than Null-EPC-CM on Decreasing Nox2 and Nox4 Expressions, Whereas ACE2-EPC-CM^EX-^ and Null-EPC-CM^EX-^ Reduced This Effect

**Figure 7:**
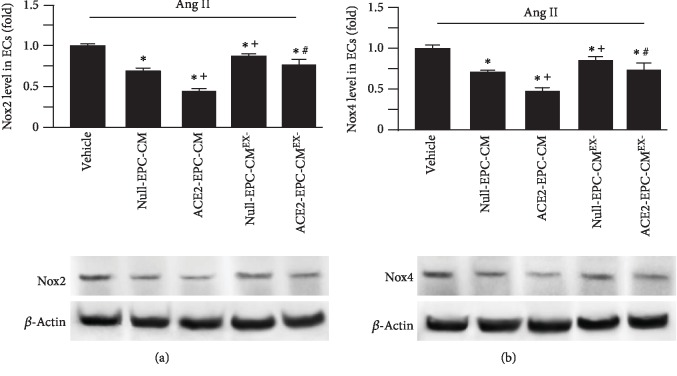
ACE2-EPC-CM decreased the expressions of Nox2 and Nox4. (a, b) Representative bands and summarized data showing the expressions of Nox2 and Nox4 in ECs exposed to Ang II. ^∗^*P* < 0.05 vs. vehicle; ^+^*P* < 0.05 vs. Null-EPC-CM; ^#^*P* < 0.05 vs. ACE2-EPC-CM. *N* = 6/group.

To explore whether oxidative stress-related proteins are involved, we measured the levels of Nox2 and Nox4 in ECs. As shown in Figure 7, Null-EPC-CM treatment significantly downregulated the expressions of Nox2 and Nox4 as induced by Ang II. This effect was remarkably enhanced by ACE2-EPC-CM, whereas it was decreased by ACE2-EPC-CM^EX-^. These data indicate that ACE2-EPC-CM had better efficacy in antioxidative stress in ECs, which might be modulated by their exosomes.

### 3.7. ACE2-EPC-EXs Were More Effective than EPC-EXs on Improving the Apoptosis and Functions of ECs from R+ Mice

**Figure 8:**
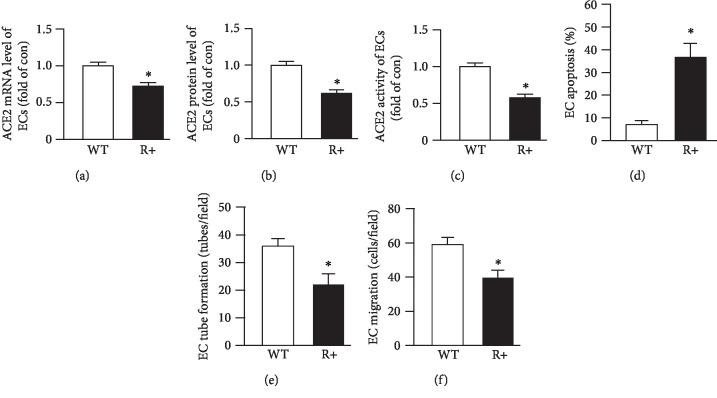
The dysfunction of ECs from R+ mice with decreased levels of ACE2 expression and activity. (a) ACE2 mRNA level of ECs. (b) ACE2 protein level of ECs. (c) ACE2 activity of ECs. (d) EC apoptotic rate is increased in R+ mice. (e, f) EC function is impaired in R+ mice. ^∗^*P* < 0.05 vs. vehicle; ^+^*P* < 0.05 vs. WT. *N* = 10/group.

To further confirm the role of ACE2-EPCs and their released EXs in ECs, R+ transgenic mice with the high plasma level of Ang-II were used. First of all, we determined the function of ECs from R+ mice and the expression and activity of ACE2. We found that the ACE2 mRNA, protein levels, and activation of ECs were decreased in the R+ mice when compared to the WT mice (Figures 8(a)–8(c)). As expected, the apoptosis of ECs of R+ mice was increased, meanwhile the tube formation and migration functions were impaired (Figures 8(d)–8(f)).

**Figure 9:**
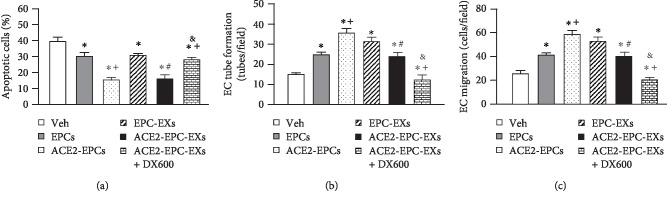
ACE2-EPC-EXs decreased apoptosis and improved the function of ECs from R+ mice. (a) The effects of ACE2-EPC-EXs on EC apoptosis. (b) The effects of ACE2-EPC-EXs on EC tube formation. (c) The effects of ACE2-EPC-EXs on EC migration. ^∗^*P* < 0.05 vs. Veh; ^+^*P* < 0.05 vs. EPCs; ^#^*P* < 0.05 vs. EPC-EXs; ^&^*P* < 0.05 vs. ACE2-EPC-EXs. *N* = 10/group.

We also performed a functional study to determine the effects of ACE-EPC-EXs on ECs from R+ mice. The results showed that ACE2-EPCs had better efficacy in attenuating the EC apoptosis and augmenting EC functions than EPCs. The ACE2-EPC-EXs had similar effects as ACE2-EPCs in those functions (Figure 9). The ACE2 inhibitor, DX600, could block the beneficial effects of ACE2-EPC-EXs. These data indicate that ACE2-EPCs provide beneficial effects on ECs, and the protective effects of ACE2-EPCs could be contributed from their released EXs (ACE2-EPC-EXs).

## 4. Discussion

In the present study, we found that ACE2-EPCs exhibited beneficial effects on protecting ECs against Ang II-induced injury and dysfunction through improving the mitochondrial function. What's more, depletion of EXs in the culture medium of ACE2-EPCs abundantly reduced these protective effects.

Increasing evidence shows that ACE2 provides vascular protective effects by counteracting the deleterious effects of Ang II and has great potential for treating vascular diseases [27, 28]. Our previous study has demonstrated that ACE2 transduction could downregulate the expressions of Nox2 and Nox4 in EPCs obtained from renin and angiotensin double transgene mice [14]. In this study, we collected the culture medium of ACE2-EPCs and found that ACE2 transduction raised the ACE2 mRNA level and the secretion of growth factors, VEGF and IGF, in EPCs.

We have previously shown that Ang II can induce cardiomyocyte hypertrophy, apoptosis, and inflammation [24]. Here, we characterized the model of Ang II-injured ECs. Except for the increased apoptosis and compromised angiogenic function, we found that the mRNA level of ACE2 of ECs was significantly reduced by Ang II stimulation. Meanwhile, we observed that the MMP of ECs was also decreased, accompanied by a larger percentage of mitochondria fragmentation as compared to the cells in the control. This is supported by a previous report showing that Ang II can induce the suppression of MMP and cellular ATP production of osteoblasts [10]. Nevertheless, whether the decreased level of ACE2 was linked with mitochondria dysfunction of ECs remains elusive. As mentioned above, the culture medium of ACE2-EPCs (ACE2-EPC-CM) harbors a high level of ACE2. Thus, to find out whether ACE2-EPCs have protective effects on Ang II-injured ECs, we treated ECs with ACE2-EPC-CM. Surprisingly, we observed that ACE2-EPC-CM exhibited antiapoptotic and proangiogenic effects (tube formation and migration abilities) on Ang II-injured ECs. According to the analysis of qRT-PCR, we found that the ACE2 mRNA level was significantly raised in ECs after ACE2-EPC-CM treatment. Consistently, the balance of Ang II/Ang (1–7) was abundantly reduced, suggesting that ACE2 carried by ACE2-EPC-CM plays a role in counteracting with Ang II.

EXs are cell-derived extracellular vesicles that could be harvested from the culture medium of cells. To date, increasing evidence indicates that the cargoes of EXs include mRNA, microRNA, and proteins. EXs participate in intercellular communication via conveying their cargoes to affect the function of recipient cells [15, 21]. Based on this information, we were wondering whether the beneficial effects elicited by ACE2-EPC-CM was ascribed to their EXs. To answer this, we depleted EXs from ACE2-EPC-CM through ultracentrifugation and then performed the coculture experiments. Our data indicate that the favorable effects offered by ACE2-EPC-CM were profoundly decreased by ACE2-EPC-CM^EX-^ (culture medium depleted of EXs). This observation supported our hypothesis that EXs contribute to the beneficial effects of ACE2-EPC-CM on Ang II-injured ECs.

Meanwhile, we did find that ACE2-EPC-CM improved Ang II-compromised mitochondrial function as revealed by increased MMP and reduced mitochondrial fragmentation. In contrast, ACE2-EPC-CM^EX-^ treatment partially decreased these protective effects. The remaining effects might be ascribed to VEGF and IGF in the culture medium. Also, we found that ACE2-EPC-CM prominently decreased Ang II-induced upregulation of Nox2 and Nox4 on ECs. Similarly, ACE2-EPC-CM^EX-^ attenuated these effects. All of these findings suggest that EXs are largely responsible for the beneficial effects of ACE2-EPC-CM through improving the mitochondria function.

To further verify the hypothesis that the beneficial effects of ACE2-EPCs are from their released EXs. We determined the protective effects of ACE2-EPCs-EXs on ECs in vivo. We used R+ mice which have a high level of Ang II in the plasma as a model. First of all, we characterized the model with the ACE2 expression level in ECs. We found that both the ACE2 mRNA and protein levels were increased in the ECs from R+ mice. This could be explained by the increased plasma level of Ang II. As we have known that ACE2 is an enzyme to catalyze the Ang II to Ang-(1–7). Hence, the increased level of Ang II could turn the activity of ACE2 up to downregulate Ang II. In addition, we found that the EC functions were impaired which agrees with our study and previous studies that Ang II induce the dysfunction of ECs [29]. More importantly, functional studies showed the beneficial effects of ACE2-EPC-EXs on ECs, which are similar to the effects that ACE2-EPCs had. These data confirmed that ACE2-EPC-EXs could protect ECs from Ang II-induced injury. The blockage of ACE2 function by its inhibitor, DX600, could abolish the protective effects of ACE2-EPC-EXs suggesting that the protective effects are from their carried ACE2.

In conclusion, ACE2-EPC-CM has protective effects on ECs against Ang II injury through the exosomal effects on decreasing apoptosis, improving angiogenic and mitochondria function.

## Data Availability

The data used to support the findings of this study are included within the article. The authors stated that the data underlying the findings of this manuscript is available to share.
